# Uterine Tumors Resembling Ovarian Sex-Cord Tumors (UTROSCTs): Case Report and Narrative Review of the Literature

**DOI:** 10.3390/jcm14051430

**Published:** 2025-02-20

**Authors:** Guglielmo Stabile, Laura Vona, Maria Carmela Pedicillo, Elisabetta Antonucci, Davide Arrigo, Marco D’Indinosante, Matteo Bruno, Tamara Stampalija, Luigi Nappi

**Affiliations:** 1 Department of Medical and Surgical Sciences, Institute of Obstetrics and Gynecology, University of Foggia, 71121 Foggia, Italy; laura.vona@unifg.it (L.V.); luigi.nappi@unifg.it (L.N.); 2 Pathology Unit, Department of Clinical and Experimental Medicine, University of Foggia, Viale L. Pinto 1, 71122 Foggia, Italy; mariacarmela.pedicillo@unifg.it (M.C.P.); antonuccielisabetta@libero.it (E.A.); 3 Dipartimento Scienze della Salute della Donna, del Bambino e di Sanità Pubblica, Fondazione Policlinico Universitario Agostino Gemelli, Instituto di Ricovero e Cura a Carattere Scientifico (IRCCS), 00136 Rome, Italy; davide.arrigo97@gmail.com (D.A.); marco.dindinosante@gmail.com (M.D.); brunomatteo2@gmail.com (M.B.); 4 Institute for Maternal and Child Health IRCCS “Burlo Garofolo”, 34100 Trieste, Italy; tamara.stampalija@burlo.trieste.it; 5 Department of Medicine, Surgery and Health Sciences, University of Trieste, 34100 Trieste, Italy

**Keywords:** uterine tumor, ovarian sex-cord tumors, UTROSCT, review, management

## Abstract

Uterine tumors resembling ovarian sex-cord tumors (UTROSCTs) are among the rarest types of uterine tumors. Diagnosis of a UTROSCT is often challenging. Imaging techniques such as ultrasound and MRI are limited in distinguishing UTROSCTs as their appearance is usually suggestive of uterine leiomyoma or adenomyosis. Additionally, the value of a preoperative biopsy remains uncertain due to the heterogeneous composition of the tumor and the inadequacy of limited samplings. We present a rare case of UTROSCT in a 59-year-old woman and we have performed a narrative review of the literature on PubMed, Scopus, and Web of Science from 2000 to June 2024, identifying 133 cases. According to our review, at histological exam UTROSCTs are mainly composed of cells resembling ovarian sex-cord elements which are arranged in cords or trabeculae, typically with a mild cytologic atypia. The most expressed sex-cord differentiation markers include inhibin, calretinin, melan A, CD56, CD99, SF1, WT1, CD10, and FOXL2. For women who have completed their reproductive plans, a total hysterectomy with adnexectomy is an adequate treatment for tumors confined to the uterus. For younger patients who wish to preserve fertility, tumorectomy via hysteroscopy or laparoscopy is the preferred treatment option and the recurrence rates range from 5% to 30%. Treatments for recurrent disease include surgery, chemotherapy, and radiation therapy, often used in combination. Advancements in molecular profiling and immunohistochemistry will improve our ability to diagnose and manage this tumor. Such investigations will enhance prognostic stratification, facilitating more accurate predictions of biological behavior and recurrence risk.

## 1. Introduction

Uterine tumors resembling ovarian sex-cord tumors (UTROSCTs) represent one of the rarest forms of uterine tumors and are uniquely classified within the group of “Mesenchymal tumors specific to the uterus” with “unspecified, borderline, or uncertain behavior” according to the WHO [1].

These tumors primarily affect women around the fifth decade of life; however, in the largest case series reported by Hurrell et al., UTROSCTs have been observed in both premenopausal and postmenopausal women, with ages ranging from 12 to 86 years [2]. These findings were further corroborated by a recent review by Watrowski et al. [3]. UTROSCTs were first described by Morehead and Bowman in 1945. Histological characteristics reproduced the ovarian sex-cord tumors’ appearance [4].

In 1976, Clement and Scully categorized UTROSCTs into two distinct types based on the proportion of sex cord-like elements (although a large portion of literature refers to UTROSCTs generally without subcategorization) [5]. The two distinct types are as follows:

Type 1: In which the epithelial-like structures that have an appearance reminiscent of ovarian sex-cord stromal tumor areas are <50% and are referred to as endometrial stromal tumors with sex cord-like elements (ESTSCLEs). These tumors have higher malignant potential and are classified as low-grade endometrial stromal sarcomas (ESSs).

Type 2: Uterine mural masses with predominant or exclusive histological appearance of sex cord elements (>50%) and a lack of stromal component and infiltrative pattern typically seen in ESSs. Known as classic UTROSCTs, these tumors generally present a low-grade malignant potential and typically exhibit benign behavior, though recurrences may occasionally occur [6].

The origin of UTROSCTs remains unclear, but it most likely arises from pluripotential uterine mesenchymal cells.

Diagnosing these tumors is particularly challenging due to their rarity and because their clinical and imaging features often resemble those of fibroids. Most cases are asymptomatic; but when symptoms occur, they typically mimic those of leiomyomas, such as postmenopausal vaginal bleeding or abnormal menstruation and pelvic pain. Diagnosis is only confirmed through postoperative histological analysis.

Both UTROSCT and ESTSCLE present as intramural, submucosal, or subserosal masses, usually well demarcated, though borders may be irregular with signs of myometrial and/or serosal infiltration, and potential involvement of the endometrium. Cervical and extrauterine spread is uncommon, occurring in less than 10% of cases. The average tumor size is approximately 6 cm, though around 20% of cases exceed 10 cm. On gross examination, these tumors often appear fleshy and range in color from gray and yellow to white [7].

The purpose of this study is to report a new identified case of UTROSCT and to provide a narrative review of the literature. By examining the clinical presentation, diagnostic approach (including immunohistochemical and molecular aspects), management strategies, and outcomes, we aim to contribute to the understanding of this rare condition.

## 2. Case Report

In June 2024, a 59-year-old patient with a BMI of 26 and a history of Hodgkin’s lymphoma, successfully treated in 2019 with chemotherapy, was referred to our center due to abnormal uterine bleeding. Blood count, coagulation, liver enzymes, and Haemoglobin appear to be in the normal range.

Transvaginal ultrasound examination by an experienced gynecologist reveals a normal anteverted uterus with regular margins and an inhomogeneous echo pattern. The endometrial thickness was normal for a postmenopausal woman. Both ovaries appeared to be regular. A well-defined oval mass measuring 39.5 × 32.2 × 35 mm occupied the posterior uterine wall. The mass showed an accentuated vascularization on color Doppler ultrasound (color score 3). No pelvic fluid was observed. The lesion imprinted the uterine cavity, presented an ultrasound appearance similar to that of a leiomyoma and was removed without macroscopic residuals with a 26Fr resectoscope (Storz resectoscope). The fragments appear whitish and also the hysteroscopic appearance was similar to that of a leiomyoma.

**Figure 1:**
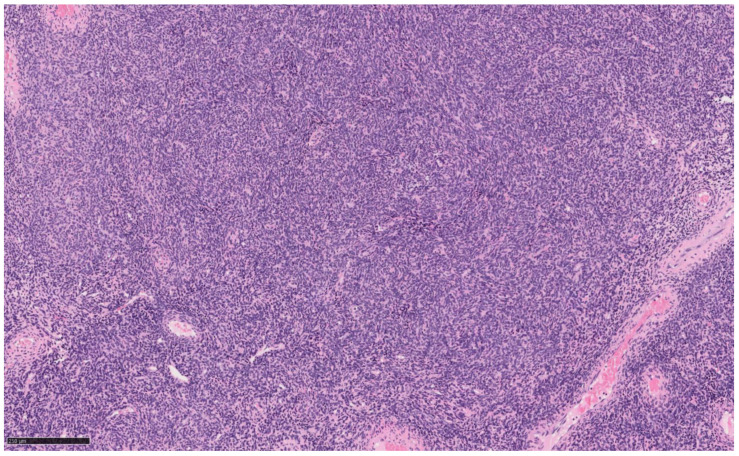
Hematoxylin-eosin magnification at 10x of uterine mass hysteroscopically removed.

Histological analysis describes a mesenchymal neoplasm consisting of a laminae of cells with uniform nuclei and inconspicuous nucleoli to which are associated bundles of smooth muscle tissue [Figure 1]. No mitotic figures and areas of coagulative necrosis were observed. On final pathological examination, the mass was diagnosed as UTROSCT.

**Figure 2:**
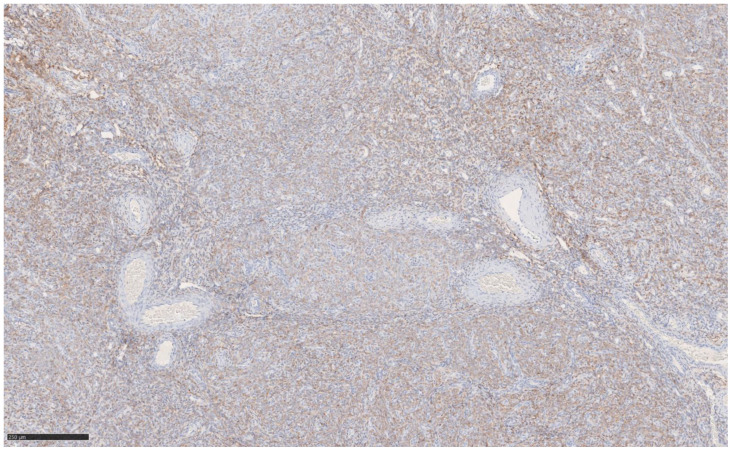
Immunohistochemistry CD56 at 10× magnification.

**Figure 3:**
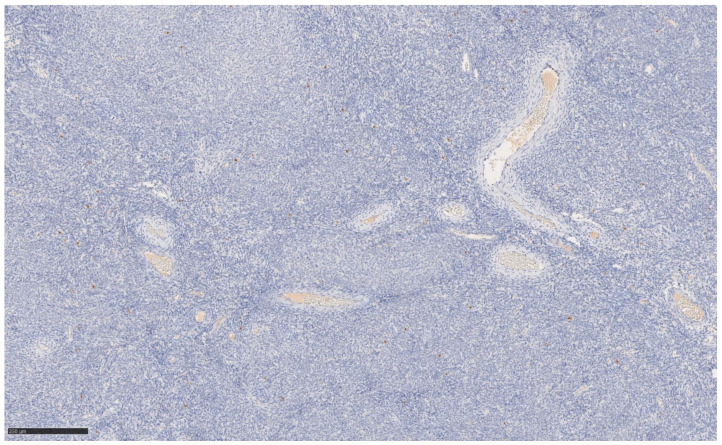
Immunohistochemistry calretinin at 10× magnification.

**Figure 4:**
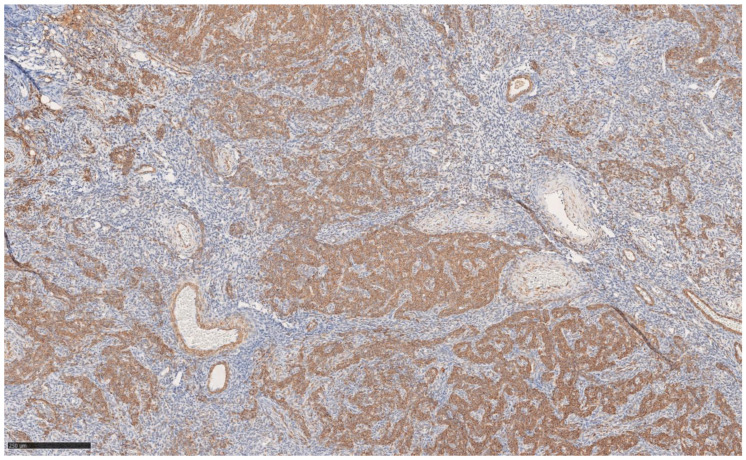
Immunohistochemistry actin at 10× magnification.

**Figure 5:**
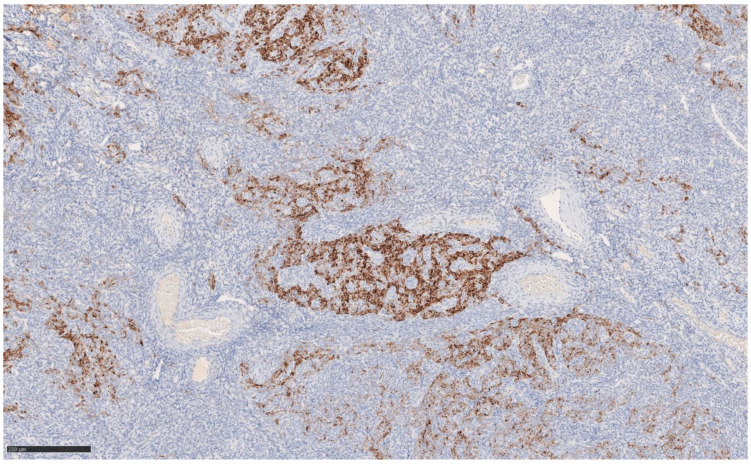
Immunohistochemistry desmina at 10× magnification.

The immunohistochemical variety found still argues for classic UTROSCT and demonstrated the following:

- Epithelial markers positivity: AE1/3.
- Sex cord marker positive: CD56 [Figure 2] and WT1; CD 10 focal positivity.
- Sex cord markers negative: calretinin [Figure 3], CD59, inhibin.
- Myoid markers positive in the bundles of smooth muscle tissue: M. Actin and desmin [Figure 4 and Figure 5].

Following the histological diagnosis in July 2024, a total-body computed tomography (CT) scan was performed, which resulted negative for metastasis.

Considering the patient’s age, a total laparoscopic hysterectomy with bilateral salpingo-oophorectomy was planned after a multidisciplinary team consultation.

The surgery was uneventful, and the patient recovered well. A follow-up ultrasound examination at six months showed no signs of recurrence.

## 3. Materials and Methods

We have performed our research on PubMed, Scopus, and Web of Science from 2000 to June 2024 to identify articles involving patients with UTROSCT. We have carried out our research since 2000, evaluating the evolution of technology in diagnosis and surgical techniques useful for the diagnosis and management of this pathology so as to compare the literature with what has been the management of our case.

The research was focused on original articles in English. We have excluded articles and manuscripts not relevant for our review and articles not in English. We have used the following keywords with a different combination of the terms: “uterine tumor resembling ovarian sex cord tumors”, “UTROSCT”, “ESTSCLE”, “sex-cord”, and “sex-cord like”. References for each article were searched to identify possible missing studies. Due to the rarity of this pathology and considering that almost all the manuscripts involved in our research are case report or case series, we present the data in a descriptive manner. For the case report we have followed the guidelines of the CARE checklist (Supplementary Table S1).

## 4. Results

In our review, we identified 118 manuscripts, reporting a total of 133 cases of UTROSCTs through the research on databases and references of the scientific papers. The mean age of the patients was 47.7 years, with cases ranging from 12 to 86 years of age.

### 4.1. Risk Factors and Clinical Presentation

Currently, there are no established risk factors for UTROSCTs, and no hereditary background has been identified. A few cases have involved patients previously treated with tamoxifen, prompting speculation of a potential causal relationship. However, the hypothesis remains weak since the majority of UTROSCTs occur independently of tamoxifen exposure, and the incidence of UTROSCT is disproportionately low compared to the number of women exposed to tamoxifen overall [8].

The most common clinical presentation, as observed in our case, was abnormal uterine bleeding (AUB), pelvic pain, and abdominal discomfort. Some cases were asymptomatic, and the tumor was incidentally diagnosed. Rarely, hormonal disturbances such as hyperprolactinemia with galactorrhea or hypercalcemia due to ectopic PTH-related peptide production have been reported [9].

In a few instances, UTROSCTs presented with distant metastases at the time of diagnosis, involving thoracic and abdominal sites such as the lungs, lymph nodes, appendix, omentum, ovaries, peritoneum, and sigmoid colon [3].

### 4.2. Diagnostic Challenges

UTROSCTs often mimic leiomyomas in their submucosal or intramural presentation, as seen in our case. This similarity frequently leads to misdiagnosis, especially since UTROSCTs often coexist with the presence of other leiomyomas, and infrequently with other types of neoplasm including ovarian sex-cord stromal tumors, or other rare gynecological and non-gynecological tumors. Even the classic markers are often unreliable, as they are frequently negative, further complicating diagnosis.

Imaging techniques, including ultrasound and MRI, are limited in trying to distinguish UTROSCTs from leiomyomas, adenomyosis, smooth muscle tumors of uncertain malignant potential (STUMP), or leiomyosarcoma. Ultrasounds hardly discern UTROSCTs from typical leiomyomas, leiomyoma variants, adenomyomas, or leiomyosarcoma [10].

In some instances, UTROSCTs may exhibit a characteristic “sponge-like” appearance, reflecting the pathologic features of ovarian granulosa cell tumor, rarely seen in the other uterine tumors, but still not pathognomonic [11]. 

Endometrial curettage frequently results in false-negative or misleading results. Additionally, the value of a preoperative biopsy remains uncertain due to the heterogeneous composition of the tumor and the inadequacy of limited samplings [2]. 

### 4.3. Histological and Immunohistochemical Features

The average tumor size was 6 cm. Most tumors presented as yellow, tan-pink, or tan-gray masses, occasionally accompanied by hemorrhage areas. At histological exam, UTROSCTs are mainly composed of cells which are arranged in cords or trabeculae, typically with a mild cytologic atypia.

UTROSCTs exhibit a polyphenotypic immunophenotype with co-expression of markers of epithelial, myoid, and sex cord lineage, along with hormone receptors.

The most expressed sex-cord differentiation markers include inhibin, calretinin, melan A, CD56, CD99, SF1, WT1, CD10, and FOXL2. Additionally, epithelial (low-weight cytokeratins, EMA) and mesenchymal (vimentin, smooth muscle actin, desmin) immunophenotypical differentiation may be present. AE1/AE3 are usually focally or occasionally diffusely positive. ER and PR often show diffuse expression [12].

According to the systematic review by Watrowksi et al., the typical immunohistochemical profile of UTROSCTs is represented by a panel comprising calretinin, inhibin, CD99, and melan A—markers indicative of sex cord lineage. Positivity for calretinin, in conjunction with at least one other marker from this panel, is diagnostic for UTROSCT, whereas ESTSCLE generally express a single sex-cord marker, predominantly calretinin; this may be due to the sex cord elements in ESTSCLE constituting a minor part of the tumor and lacking the great variety of histological features observed in UTROSCT [3].

According to Özer et al., the immunoreactivity of CD56 has not been systematically studied in UTROSCs and has therefore only recently been accepted as an immunohistochemical marker of cord differentiation in addition to calretinin, inhibin, CD99, and melan A. In fact, in their case report calretinin was negative and CD56 and WT1 were positive, as in our case [12].

Moreover, a review on the immunohistochemical features of the 44 cases of UTROSCT reported by Abdullazade et al. [13] shows calretinin in 94%, inhibin expression in 49%, caldesmon in 7%, desmin in 46%, AE1/AE3 in 73%, EMA in 29%, CD10 in 50%, and CD56 in 100% of the patients.

In light of what was reported by Abdullazade and according to the review of Lin et al. both CD56 and calretinin should be used for the differential diagnosis of UTROSCTs [9]. Such as in our patient, in the case series, and the literature review, CD56 as well as WT1 turn out to be positive in all patients tested and one case was negative for calretinin.

In conclusion, it is true that calretinin is the most widely used marker for the diagnosis of UTROSC, but other markers should also be considered.

### 4.4. The Role of Molecular Profiling

The emergence of increasing reports of disease recurrence has led to a reconsideration of UTROSCT as a tumor with low malignant potential. Blake et al. identified a recurrence rate of nearly 5.5%, but it was reported up to 30% in various studies [7]. 

Furthermore, the low incidence and broad histologic spectrum of UTROSCTs make them difficult to be diagnosed. For these reasons, recent studies focused on a better understanding of UTROSCTs’ typical molecular markers and on the identification of valuable prognostic factors especially in the subset of aggressive cases by exploring molecular characteristics and genetic profiling.

Next-generation sequencing (NGS) and RNA-sequencing allowed for the identification of several molecular abnormalities involved in the pathogenesis of UTROSCTs, primarily including abnormal translocation of the nuclear receptor coactivator (NCOA) gene family, having either estrogen receptor 1 (ESR1) or growth regulating estrogen receptor binding 1 (GREB1) as partner genes.

UTROSCTs also typically lack molecular alterations found in other tumors, such as in ESTSCLEs (JAZF1-SUZ12 gene fusion and PHF1 rearrangement) [2,14,15].

Analysis of the tumor microenvironment and gene molecular subtyping through FISH or RNA-sequencing may be valuable in predicting tumor behavior and prognosis, as well as aiding in the differential diagnosis of tumors with similar histology but distinct molecular profiles. These include endometrial cancer with sex cord differentiation, endometrial stromal tumors with sex cord elements, adenosarcoma with widespread sex cord components, and epithelioid smooth muscle tumors. Of note, the placement of tumors with sex cord-like differentiation is very dependent on accurate sampling of the neoplasm, which is not possible in a biopsy or in curettage specimens. Genetic profiling may provide additional benefit, whether in such cases or when histological features are not well defined. In summary, immunohistochemical expression of sex cord markers (inhibin, calretinin, SF1, FOXL2) and/or detection of GREB1 or ESR1 fusions through FISH (NCOA1, NCOA2, NCOA3) is confirmatory for a differential diagnosis [15,16,17,18,19].

### 4.5. Radical and Fertility-Sparing Treatment Strategies

In women who have completed their reproductive plans, a hysterectomy with adnexectomy is an adequate treatment for tumors confined to the uterus. In premenopausal patients, the preservation of the ovaries may be considered. Pelvic lymphadenectomy is not routinely recommended and should only be performed when bulky lymph nodes are present. For younger patients who wish to preserve fertility, tumorectomy via hysteroscopy or laparoscopy is the preferred treatment option. Among patients who underwent a fertility sparing approach with uterine preservation, recurrence rates do not appear to be significantly higher compared to those who underwent more radical procedures, and reproductive outcomes have been favorable. To date, several cases of pregnancy have been reported in women undergoing fertility sparing treatment for UTROSC [3,20].

### 4.6. Recurrences: Diagnosis and Therapeutic Approach

Recurrences are typically detected via ultrasound or MRI. While no specific serum tumor markers have been validated, any markers that were elevated at the moment of diagnosis and responded to therapies should be monitored during follow-up. In some cases, an increase in serum CA125 has been reported prior to recurrence [21]. Cömert et al. [22] estimate the average recurrence rate of UTROSCT to be 6.3%. Regarding the follow-up, De Franciscis et al. suggest transvaginal ultrasound every six months; however, follow-up protocols for UTROSCT differ among authors and a 3- to 5-year length is suggested [3,23]. In cases managed conservatively, repeat hysteroscopy may be warranted to exclude residual tumor or as part of ongoing surveillance [23].

In case of recurrence, the most effective treatment is represented by radical surgery with the complete removal of the disease. The response to chemotherapy and hormonal treatments is generally poor (Supplementary Table S2).

## 5. Discussion

UTROSCT is an extremely rare mesenchymal neoplasm with uncertain origins, primarily named for its morphological similarity to ovarian sex-cord tumors. It is generally considered of low malignant potential, typically diagnosed at an early stage.

UTROSCTs mainly affect in women in the fifth decade, often present with irregular vaginal bleeding or chronic pelvic pain as observed in our case. Approximately 20% of patients are under 40 years at the diagnosis. Imaging techniques including ultrasound and MRI have limited ability to distinguish UTROSCTs as their appearance is varied and often mimics more common uterine pathologies like leiomyomas or adenomyosis. Hysteroscopic evaluation similarly lacks specificity as UTROSCTs may present with images resembling those of classic leiomyomas, often with accentuated vascularization, further confounded by the frequent coexistence of leiomyomas.

Diagnosis of a UTROSCT is based upon morphologic features on Hematoxylin and Eosin staining, confirmed by immunohistochemical staining. The cells have variable features, including epithelioid and spindle shaped cells and bland, uniform cells with scant to abundant eosinophilic or foamy cytoplasm. They arrange in different architectural patterns, including diffuse, nested, corded, tubular, and retiform growth reminiscent of sex-cord stromal tumors. In fact, they morphologically resemble an ovarian sex-cord tumor, although in most cases it does not precisely resemble any of the specific types of neoplasia in this category [24].

UTROSCTs and ESTSCLEs characteristically exhibit a polyphenotypic immunophenotype with co-expression of epithelial, myoid, and sex cord markers. For this reason, in 2008, Czernobilsky introduced diagnostic criteria based on immunohistochemical markers [25]. The sex cord markers calretinin, inhibin, CD99, and melan A, have been recognized as the most characteristic markers for confirming the diagnosis of UTROSCT, and positivity for calretinin and for at least one of the other three markers in this panel is highly suggestive of type 2 tumors [3]. However, as shown by several studies, calretinin might be negative in some cases of UTROSC, while other sex cord markers such as CD56, CD10, and WT1 will be positive, as in our case. For example, CD56 seems to be a more sensitive marker but has not been systematically studied in UTROSCs and has therefore only recently been accepted as an immunohistochemical marker of cord differentiation in addition to calretinin, inhibin, CD99, and melan A [12,13].

Moreover, UTROSCTs display a much broader range of positive immunohistochemical markers including epithelial (AE1/3, epithelial membrane antigen) and myoid (desmin, a smooth muscle actin, h-caldesmon) markers, which were positive in our histological exam. Also, neuroendocrine markers (chromogranin), hormone receptors (estrogen receptor, progesterone receptor, androgen receptor), vimentin, and HMB45 are frequently expressed. It should also be noted that these markers are not specific to cord differentiation and may only be found in isolated cases with varying degrees and intensities.

Surgical intervention remains the cornerstone of UTROSCT management, with hysterectomy—with or without salpingo-oophorectomy—being the primary treatment option for middle-aged or older women. In our case, considering the patient’s prior oncological history, we opted for a hysterectomy with bilateral salpingo-oophorectomy. However, since approximately 20% of patients are aged <40 years at diagnosis, fertility preservation treatment should also be considered. Conservative approaches, such as hysteroscopic or laparoscopic tumorectomy, have demonstrated favorable outcomes with acceptable recurrence rates and successful pregnancies reported [9,26,27].

The prognosis for UTROSCT is generally favorable. A study by Blake et al. [7] reported overall survival rates of 97% at 1-, 2-, and 5-years post-surgery, with no significant differences between total abdominal hysterectomy alone and combined hysterectomy with adnexectomy. Adjuvant treatments, including chemotherapy (platinum-based compounds as standard treatment for ovarian cancer, or bleomycin, etoposide, and cisplatin schedule based on sex-cord stromal tumors of the ovary), hormone therapy (such as megestrol and letrozole), and radiotherapy, remain controversial and are typically reserved for cases with high recurrence or metastatic risk. In our patient, no adjuvant therapy was administered due to the absence of high-risk features

Recurrence rates for UTROSCT range from 5% to 30%, with the pelvis, abdominal cavity, and vagina being the most common sites of recurrence. Rarely, distant recurrences involving the lungs, liver, and vertebrae have been reported [9,26].

Notwithstanding the mildly higher recurrence rate, there is no reported significant difference in 5 year and 10 year DFS in patients who underwent conservative mass resection compared to radical surgery with hysterectomy [28]. Factors associated with lower disease-free survival (DFS) include pelvic pain at diagnosis, type I disease, tumor size ≥ 10 cm, cervical or extrauterine disease, and lymphovascular space invasion. None of these factors were present in our case, supporting our decision for a surgical approach without adjuvant therapy [2]. However, predictive factors for disease recurrence remain poorly understood due to the limited number of cases and appropriate follow-up data available in the literature. Regarding the latter, imaging modalities used for diagnosis, such as transvaginal ultrasonography, are suggested to be performed twice a year, for 3 to 5 years [3]. However, the extremely limited number of cases described does not allow for meaningful treatment recommendations to be established [28].

The strength of our case lies in the fact that it is one of the few in the literature to have an immunohistochemical profile with negative calretinin and positive CD56 and WT1. Furthermore, the strength of our review is represented by the long period of time overviewed in the literature and our analysis on the role of molecular profiling.

The main limitation of our review is represented by the presence in the literature of almost only case reports. This does not allow for an in-depth analysis of the data which can be altered by numerous biases.

## 6. Conclusions

UTROSCTs are rare and diagnostically challenging tumors. Advancements in molecular profiling and immunohistochemistry will improve our ability to diagnose and manage these tumors. The integration of molecular diagnostics, alongside traditional histopathology and immunohistochemistry, allows for distinguishing UTROSCTs from other uterine tumors with sex cord differentiation. Such investigations will enhance prognostic stratification, facilitating more accurate predictions of biological behavior and recurrence risk, thereby contributing to the optimization of personalized therapeutic strategies, although this tumor seems to respond well to conservative treatment in most cases, with a low risk of recurrence.

## Data Availability

The original contributions presented in the study are included in the article. Further inquiries can be directed to the corresponding author.

## Supplementary Materials

The following supporting information can be downloaded at: https://www.mdpi.com/article/10.3390/jcm14051430/s1, Supplementary Table S1. CARE Checklist; Supplementary Table S2. Manuscript considered for the analysis.
