# Supplementary material for: Uterine Tumors Resembling Ovarian Sex-Cord Tumors (UTROSCTs): Case Report and Narrative Review of the Literature
Source: J Clin Med. 2025 Feb 20;14(5):1430. doi: 10.3390/jcm14051430 (PMC11901091; doi:10.3390/jcm14051430)
Supplement: Supplementary file 1 [file jcm-14-01430-s001.zip › jcm-3389534-supplementary.pdf]

CARE case report guidelines

# CARE Checklist of information to include when writing a case report

(cc) BY-NC-ND

| Topic | Item | Checklist item description | Reported on Line |
|---|---|---|---|
| Title | 1 | The diagnosis or intervention of primary focus followed by the words "case report" | Line 1-3 |
| Key Words | 2 | 2 to 5 key words that identify diagnoses or interventions in this case report, including "case report" | Line 32 |
| Abstract (no references) | 3a | Introduction: What is unique about this case and what does it add to the scientific literature? | Line 20-24 |
| | 3b | Main symptoms and/or important clinical findings | Line 20-24 |
| | 3c | The main diagnoses, therapeutic interventions, and outcomes | Line 25-27 |
| | 3d | Conclusion—What is the main "take-away" lesson(s) from this case? | Line 27-32 |
| Introduction | 4 | One or two paragraphs summarizing why this case is unique (**may include** reference**s**) | Line 74-78 |
| Patient Information | 5a | De-identified patient specific information | Line 80-81 |
| | 5b | Primary concerns and symptoms of the patient | Line 82-84 |
| | 5c | Medical, family, and psycho-social history including relevant genetic information | Line 80-81 |
| | 5d | Relevant past interventions with outcomes | Line 80-81 |
| Clinical Findings | 6 | Describe significant physical examination (PE) and important clinical findings | Line 84-90 |
| Timeline | 7 | Historical and current information from this episode of care organized as a timeline | N.A. |
| Diagnostic Assessment | 8a | Diagnostic testing (such as PE, laboratory testing, imaging, surveys). | Line 90-93 |
| | 8b | Diagnostic challenges (such as access to testing, financial, or cultural) | N.A |
| | 8c | Diagnosis (including other diagnoses considered) | Line 94-97 |
| | 8d | Prognosis (such as staging in oncology) where applicable | N.A. |
| Therapeutic Intervention | 9a | Types of therapeutic intervention (such as pharmacologic, surgical, preventive, self-care) | Line 107-108 |
| | 9b | Administration of therapeutic intervention (such as dosage, strength, duration) | Line 108-110 |
| | 9c | Changes in therapeutic intervention (with rationale) | N.A. |
| Follow-up and Outcomes | 10a | Clinician and patient-assessed outcomes (if available) | Line 108-110 |
| | 10b | Important follow-up diagnostic and other test results | Line 110 |
| | 10c | Intervention adherence and tolerability (How was this assessed?) | N.A. |
| | 10d | Adverse and unanticipated events | N.A. |

| | | |
|---|---|---|
| **Discussion** report | **11a** | A scientific discussion of the strengths AND limitations associated with this case N.A.___ |
| | **11b** | Discussion of the relevant medical literature **with references** Line 261-332 ______ |
| | **11c** | The scientific rationale for any conclusions (including assessment of possible causes) Line 326-332 ______ |
| | **11d** | The primary "take-away" lessons of this case report (without references) in a one paragraph conclusion Line 333-342 ______ |
| **Patient Perspective** | **12** | The patient should share their perspective in one to two paragraphs on the treatment(s) they received . . . . N.A. |
| **Informed Consent** | **13** | Did the patient give informed consent? Please provide if requested . . . . . . . . . . . . . . . . . . . . . . . . . . . . . . . . . . . . . . **Yes** X☐ **No** ☐ |

**Table S1. CARE Checklist**

| Author | Patients (n) | Age | Imaging | Macroscopic features | Microscopic features | Immunohistochemical features | Genetic features | Treatment |
|---|---|---|---|---|---|---|---|---|
| **Our Study** | 1 | 59 | Normal anteverted uterus with regular margins and an inhomogenous echo pattern. The endometrial thickness was normal for a postmenopausal woman. Both ovaries appeared regular. A well-defined oval mass measuring 39.5 x 32.2 x 35 mm occupied the posterior uterine wall. The mass showed an accentuated vascularisation on Colour Doppler ultrasound (Colour Score 3). No pelvic fluid was observed. The lesion imprinted the uterine cavity | The tumor fragments appear whitish and the hysteroscopic appearance was similar to that of a leiomyoma | Laminae of cells with uniform nuclei and inconspicuous nucleoli to which were associated bundles of smooth muscle tissue. No mitotic figures and areas of coagulative necrosis were observed. | Epithelial markers positivity: AE1/3<br><br>Sex cord marker positive: CD56 and WT1; CD 10 focal positivity<br><br>Sex cord markers negative: Calretinin, CD59, Inhibin<br><br>Myoid markers positive in the bundles of smooth muscle tissue: M. Actin and Desmin | NA | Total laparoscopic hysterectomy with bilateral salpingo-oophorectomy |
| **Hatice Özer et al. [12]** | 1 | 38 | US: enlarged uterus consisting of cystic and solid parts. Thin and thick septations in the heterogenous areas; some fluid image. CT: lobulated semisolid mass of 21x18x13 cm, thick-walled, with centrally intensive content | The uterus measured 25x18x8.5 cm. Intramural soft nodule of 18 cm in diameter with no connection either the endometrium or the endocervical mucosa. The tumor has a well-circumscribed contour. The cut surface partly cystic, necrotic and hemorrhagic with areas of yellowish to tan colored solid fleshy nodules. There were also intramural and subserous leiomyomas | The tumor has noninfiltrative margins. There were cystic spaces, infarct type of necrosis. The tumor cells have uniform, small, darkly staining round or oval nuclei with granular chromatin and inconspicuous nuclei that closely resemble proliferative-phase endometrial cells. Mitotic activity is about 0-1/10HPF. These cells grow in cords, trabeculae, and occasional glandular structure, some of like Call-Exner bodies. There were bundles of myoid cells within the tumor, and focal areas of clear cells. Scattered nuclear pleomorphism were only seen around the necrotic and degenerated cells. | CD56: Diffuse positive<br>CD10: Focal positive (some areas)<br>Actin, Desmin: Focal positive (some areas and myoid cells)<br>Inhibin: Focal positive<br>Estrogen receptor: Positive<br>Progesterone receptor: Positive<br>WT1: Focal positive (some areas)<br>HMB 45 : Focal positive (only myoid cells)<br>CD99; Calretinin; Pancytokeratin; CD34; CD117; S100: Negative | NA | Total abdominal hysterectomy |

| | | | | | | | | |
|---|---|---|---|---|---|---|---|---|
| **Shan Ye et al. [20]** | 5 | Early 50s; Early 60s; Early 60s; Nearly 40 years; Nearly 40 years | US: Hypoechoic area on the posterior wall of the uterus<br>with protrusion into the uterine cavity<br><br>Hypoechoic protrusion on right uterine wall with clear<br>boundary<br><br>Not available<br><br>Hypoechoic intrauterine nodules<br><br>Consider endometrial polyps | Intramural nodules protruding into the uterine cavity, 4.5 cm in diameter<br><br>Intrauterine polypoid masses, 5 cm in diameter<br><br>Intramural nodules with polypoid bulging of the uterine cavity, 4 cm in diameter<br><br>Submucosal elevation, 3 cm in diameter (fragment tissue)<br><br>Polypoid masses, 1.5 cm in diameter | Muscular invasion: 1 Tonguelike infiltration; 4 Focal infiltration<br><br>2 had Nucleolus and 3 none<br><br>Only one had Necrosis<br><br>Mitotic count (/10 HPF) one had 2; two had 1 and 2 had 0. | Case 1<br><br>CK 1+<br>CD99 2+<br>CR 2+<br>SMA 2+<br>h-Caldesmon 2+<br>BCL-2 3+<br>ER 1+<br>PR 3+<br>CD10 1+<br><br>Case 2<br><br>CK 1+<br>CD99 2+<br>CR 2+<br>$\alpha$-Inhibin 1+<br>SF-1 2+<br>Desmin 2+<br>SMA 1+<br>h-Caldesmon 2+<br>BCL-2 3+<br>ER 1+<br>PR 3+<br>CD10 1+<br><br>Case 3<br><br>CK 1+<br>WT-1 3+<br>CD99 2+<br>CR 2+<br>SF-1 2+<br>SMA 1+<br>h-Caldesmon 2+<br>BCL-2 3+<br>ER 2+<br>PR 3+<br>CD10 1+<br><br>Case 4<br><br>CK 2+<br>CD99 2+<br>Desmin 2+<br>SMA 3+<br>h-Caldesmon 2+<br>BCL-2 3+<br>ER 3+<br>PR 3+<br>CD10 1+<br><br>Case 5<br><br>CK 2+<br>WT-1 2+<br>CR 2+<br>SF-1 2+<br>Desmin 1+<br>h-Caldesmon 1+<br>BCL-2 3+<br>ER 2+<br>PR 2+ | NCOA1 detected in cases 1, 2 and 3. NCOA3 gene breaks was detected in case 4.<br>Case 5 did not have any of these gene breaks.<br>NCOA2, JAZF1 and PHF1 gene breaks were not detected in any of the five patients.<br>Somatic pathological mutations were detected in three patients.<br>FANCE gene Exon 1 pW19X stop-gain mutation (G–A) was detected in case 4.<br>The ATR gene Exon 9 pI710Yfs5 frameshift mutation (AT–A) was detected in case 3.<br>The ARID1A gene Exon 1 pP21del non-frameshift mutation (CCCG–C) was detected in case 2. | In 4 cases: hysterectomy and bilateral adnexectomy; 1 case had electrical resection |
| **Rafal Watrowski et al. [29]** | 1 | 22 | US: oval, well-demarked intrauterine mass measuring 21.7  13.0 18 mm, presumed as grade-1 submucous myoma or polyp | NA | Epitheloid neoplasm growing in trabeculae and tubules intermingled with glomerulum-like structures and showing an infiltration into the adjacent myometrial layer. The tumour cells had ovoid nuclei with finely grained chromatin and a prominent, excentrically located nucleolus. The cytoplasm of the neoplastic cells was abundant, eosinophilic and of foamy structure. The tumor cells showed 4 mitoses/10 high-power field | Calretinin Positive<br>CD99 Positive<br>CD56 Microfocally positive<br>Melan A Microfocally positive<br>Inhibin Negative<br>Vimentin Positive<br>WT1 Focally positive<br>CK 1/3 Focally positive<br>CK 7 Focally positive<br>CK 19 Focally positive<br>Desmin Focally positive<br>Estrogen receptor 75% Positive<br>Progesterone receptor 100% Positive | NA | Resectoscopic treatment |

| | | | | | | | | |
|---|---|---|---|---|---|---|---|---|
| **Rafał Watrowski et al. [3]** | 2 | 31 and 58 | Case 1<br><br>US: Well-defined, oval-shaped mass measuring 27.8 × 26.5 × 19 mm. The mass showed non-uniform echogenicity due to the presence of some anechoic cystic areas. Moderate edge shadowing was present. On color Doppler ultrasound, the lesion appeared not to be richly vascularized. No pelvic fluid was observed.<br><br>Case 2<br><br>two fibroids located in the anterior and posterior uterine wall, measuring 3.5 cm and 5 cm, respectively, and an endometrial thickness of 8 mm (despite the patient's postmenopausal status). | Case 1<br><br>Uterine posterior mass of about 30 mm<br>Area of suspicion was identified during an hysteroscopy on the anterior wall of the endometrium, measuring 1.5 cm, characterized by an irregular, yellow-colored surface. Presence of diffuse hypervascularization.<br><br>Case 2<br><br>Stretched and branched endometrial glands, surrounded by cells with abundant and foamy cytoplasm, suggesting the presence of sex cord-like elements | Case 1<br><br>Hypercellular tumor with a solid growth pattern and focal glandular and trabecular differentiation. The cells were small to medium in size, with scant cytoplasm and regular, ovoid nuclei. Mitotic activity was low, with approximately 2 mitoses per 10 high-power fields, and there was no evidence of necrosis. An infiltrative growth pattern into the myometrium was apparent, and this was suggestive of lymphovascular space invasion.<br><br>Case 2<br><br>NA | Case 1<br><br>Tumor cells positivity for ER, PR, WT1, Calretinin, CD56, CD99, Smooth Muscle Actin, and Desmin, with focal positivity for E-cadherin and p16.<br>The tumor was negative for Cyclin D1, BCOR, EMA, CK7, TTF1, GATA3, Chromogranin, Synaptophysin, Caldesmon, Cathepsin k, and Inhibin<br><br>Case 2<br><br>Tumor cells positivity for ER, PR, E-Cadherin, Design, Alpha-SMC, Inhibit; CD99, Calretinin.<br>The tumor was negative for Cathepsin K, Synaptophysin, Chromogranin, GATA3, TTF1, BCOR, Cyclin D1, P16, Caldesmon, EMA, CK7. | NA | Laparoscopic total hysterectomy with bilateral salpingo-oophorectomy |

| | | | | | | | | |
|---|---|---|---|---|---|---|---|---|
| **Samir Abdullazade et al. [13]** | 3 | 46; 30; 42 | Case 1<br><br>US: Enlarged uterus with a 10 cm diameter myometrial mass<br><br>Case 2<br>NA<br><br>Case 3<br><br>US: Uterine 1,5 cm well-circumscribed mass protruding into the uterine cavity | Case 1<br><br>The uterine wall was 4.3 cm in thickness and harbored multiple leiomyomatous nodules. Among these, a well-circumscribed, yellow-colored, 2-cm, submucosal mass was noted.<br><br>Case 2<br>NA<br><br>Case 3<br><br>1.5-cm, well-circumscribed, yellow-colored, polypoid mass arising from the myometrium | Case 1<br><br>Well-circumscribed nodular tumor situated in the myometrium without any involvement of the endometrium. No evidence of myometrial invasion. Mainly composed of epithelioid cells forming trabecular and solid patterns. The tubules forming trabecules consisted of cuboidal epithelioid cells with round nuclei, some with small nucleoli. Mitotic activity was rare (<1 per 10 high-power fields). There was neither necrosis nor vascular invasion.<br><br>Case 2<br><br>Cords and nests of epithelioid cells within a fibrous stroma; furthermore, some areas displayed an alveolar arrangement. Besides epithelioid morphology, some neoplastic cells exhibited clear cytoplasm, resembling clusters of foamy macrophages. No cellular atypia, lymphovascular invasion, infiltration, or necrosis. Mitotic activity was rarely noted (<1 per 10-high power areas).<br><br>Case 3<br><br>Epithelioid cells with inconspicuous nucleoli, arranged in anastomosing cords/tubules and nests within a fibrous stroma, resembling sex cord-stromal tumors of the ovary. No significant cellular atypia, necrosis, vascular invasion, or mitotic activity was noted. | Case 1<br><br>Tumor cells positivity for CD56, Calretinin, Desmin, AE1/AE3.<br><br>The tumor was negative for Inhibin, Caldesmon, EMA, CD10<br><br>Case 2<br><br>Tumor cells positivity for Inhibin.<br><br>The tumor was negative for AE1/AE3, EMA<br><br>Case 3<br><br>Tumor cells positivity for CD56, Calretinin, Inhibin, Desmin, AE1/AE3,<br><br>The tumor was negative for Caldesmon, EMA, CD10. | NA | 2 patients had a total abdominal hysterectomy and a bilateral salpingo-oophorectomy and one the mass resection |

**Table S2 Supplementary. Manuscript considered for the analysis**
